# Ultrasound-induced protein-polysaccharide complexes: Mechanisms, functionalities and applications in food systems^☆^

**DOI:** 10.1016/j.ultsonch.2026.107997

**Published:** 2026-08-10

**Authors:** Qiufang Liang, Panpan Cao, Yunxia Du, Haile Ma, Abdur Rehman, Ka-Hing Wong, Ren-You Gan, Mingming Zhong, Yufan Sun, Xiaofeng Ren

**Affiliations:** a School of Food and Biological Engineering, Jiangsu University, 301 Xuefu Road, Zhenjiang, Jiangsu 212013, China; b Institute of Food Physical Processing, Jiangsu University, 301 Xuefu Road, Zhenjiang, Jiangsu 212013, China; c Department of Food Science and Nutrition, The Hong Kong Polytechnic University, Hung Hom, Kowloon, Hong Kong Special Administrative Region of China; d Research Institute for Future Food, The Hong Kong Polytechnic University, Hung Hom, Kowloon, Hong Kong Special Administrative Region of China

**Keywords:** Acoustic cavitation, Non-covalent interaction, Maillard reaction, Techno-functional properties, Bioactive delivery, Emulsion stability

## Abstract

Understanding the interaction between proteins and polysaccharides is fundamental for designing food structures, yet the potential of ultrasound to precisely modulate these interactions has not been systematically reviewed. This review critically examines the mechanistic role of acoustic cavitation and its associated physical and chemical effects in driving the formation of protein-polysaccharide complexes. We highlight how ultrasound-induced shear forces, micro-turbulence, and free radical generation not only accelerate covalent conjugation via the Maillard reaction but also reinforce non-covalent forces, such as hydrophobic interactions, hydrogen bonding, and electrostatic contacts, by inducing conformational changes in biopolymers. These ultrasound-mediated structural modifications result in significantly improved techno-functional properties, including solubility, emulsification, and foaming performance. We further discuss how these enhanced complexes are enabling innovations across emulsion stabilization, gel fabrication, bioactive compound encapsulation, and edible film formation. Finally, we identify critical research priorities, particularly the correlation between ultrasonic parameters and molecular interaction mechanisms, the structural characterization of conjugates, and the evaluation of their safety for food applications. By providing a comprehensive framework that bridges fundamental sonochemistry with food material science, this review positions ultrasound as a versatile and sustainable tool for engineering advanced food systems.

## 1 Introduction

Proteins and polysaccharides constitute two essential classes of biological macromolecules in food systems. Natural proteins, predominantly sourced from animal-derived products (e.g., meat, fish, dairy) and plant-based materials (e.g., legumes, nuts), serve dual roles as nutrient providers and functional modulators [1]. Their unique three-dimensional conformations govern physicochemical properties that directly influence textural characteristics, flavor retention, and processing stability. Polysaccharides, which are complex carbohydrates composed of monosaccharide units linked by glycosidic bonds, are primarily extracted from diverse sources such as cereal grains, tubers, and fruits [2]. These carbohydrate polymers have gained prominence in food science due to their multifunctional applications as well as acting as fat mimetics, stabilizers, viscosity modifiers, gelling agents, and emulsifiers, which are attributed to their tunable molecular interactions and hydration-dependent behaviors. Fundamentally, the majority of foods are composed of diverse food components that form intricate systems, such as interactions between proteins and lipids, proteins and carbohydrates, as well as proteins and polyphenols.

**Fig. 1:**
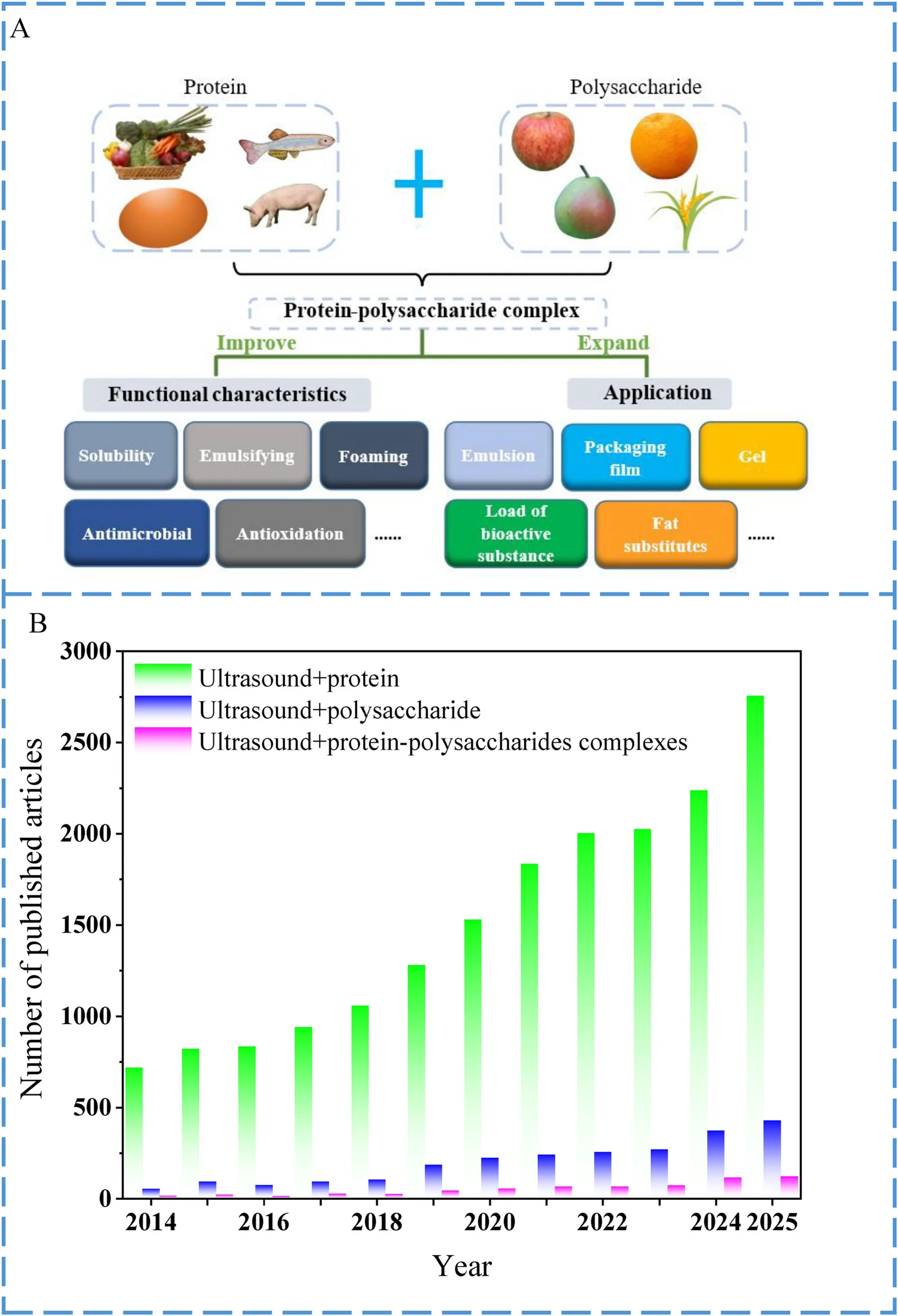
(A) Schematic overview of the primary sources of proteins and polysaccharides, and the enhanced functional properties and diverse applications of their complexes. (B) Annual publication trends from 2015 to 2024 on ultrasound-treated proteins, polysaccharides, and their binary complexes (data sourced from the Science Direct database).

Recent investigations indicate that protein-polysaccharide complexes not only preserve the nutritional attributes of their constituent macromolecules but also demonstrate enhanced functional characteristics compared to individual components (Fig. 1A) [1], [3]. These complexes play a crucial role in controlling the macroscopic properties of food, such as stability, texture, and flavor, and are instrumental in developing delivery systems for bioactive compounds [4], [5], producing edible packaging [6], and supporting biological functions [7]. The formation of these complexes relies on various intermolecular interactions, including covalent bonds, electrostatic forces, hydrophobic interactions, hydrogen bonding, and van der Waals forces, which are influenced by factors such as biopolymer concentration, pH, and ionic strength [8]. Therefore, it is essential to develop efficient strategies for precisely manipulating the interactions between proteins and polysaccharides to fabricate multi-functional complexes.

The emergence of interdisciplinary approaches has promoted the application of advanced physical processing technologies, including ultra-high pressure [9], microwave [10], cold plasma [11] and ultrasound [12], into food research. Among these, ultrasound stands out as an eco-friendly, efficient, and versatile technology that has been extensively applied in extraction [12], enzymatic hydrolysis [13] and fermentation [14]. Substantial evidence indicates that ultrasound can modify the structural characteristics of various proteins and polysaccharides, such as soy protein isolate (SPI) [15], β-lactoglobulin [16], pectin [17] and starch [18], thereby modulating their physicochemical and techno-functional properties. Furthermore, bibliometric analysis of Web of Science literature over the past decade (Fig. 1B) reveals a consistent growth in publications related to ultrasound-treated proteins, polysaccharides, and their binary complexes, reflecting increasing recognition of this field. However, studies focusing on individual components significantly outnumber those dedicated to binary composite systems. This disparity not only highlights a notable research gap but also underscores the considerable potential for investigating ultrasound-mediated protein-polysaccharide complexes. Despite the growing interest in this area, the existing literature remains fragmented. Most reviews have focused either on individual biopolymers [2], [19] or conventional complexation methods [7]. Consequently, the specific role of ultrasound in modulating protein-polysaccharide interactions has been systematically underexplored, and to the best of our knowledge, no comprehensive review has summarized the mechanisms, functional properties, and applications of ultrasound-induced complexes. This critical gap is explicitly highlighted in the comparative overview provided in Table 1, which delineates the unique contributions of this review against the backdrop of earlier studies.

**Table 1:** Comparative overview of this review versus earlier related reviews.

Aspect	Coverage in Existing Reviews	Novelty & Necessity of This Review
Scope	Focused on individual biopolymers (proteins or polysaccharides) or general complexation methods [7], [20].	Systematically focuses on ultrasound-induced binary protein-polysaccharide complexes.
Mechanisms	Fragmented coverage; ultrasound's role in intermolecular forces rarely detailed [21], [22].	Comprehensively details ultrasound's effects on both covalent and non-covalent interaction pathways.
Functional properties	Broad overview of techno-functional properties without ultrasound-specific analysis [1], [7].	Parameter-performance correlations in emulsions, gels, encapsulation, and films.
Application focus	General applications in food systems; limited linkage to ultrasound processing [1], [23].	Advanced applications in emulsions, gels, encapsulation, and edible films enabled by ultrasound.
Future Outlook	Generic recommendations for future research [1].	Specific, prioritized research directions (e.g., parameter-function correlation, safety, scale-up).

Therefore, this review aims to address this gap by elucidating the mechanistic pathways through which ultrasound promotes both covalent and non-covalent interactions between proteins and polysaccharides. It systematically details the effects of ultrasound on the functional properties of the resulting complexes, such as solubility, emulsification, and foaming capacity, and explores their advanced applications in emulsions, gels, bioactive compound encapsulation, and packaging films. Finally, specific future research priorities are outlined to guide the development of innovative and sustainable food systems based on ultrasound-assisted protein-polysaccharide complexes.

## 2 Underlying mechanisms of ultrasound in the formation of protein-polysaccharide complexes

### 2.1 Acoustic cavitation, mechanical and chemical effects

**Fig. 2:**
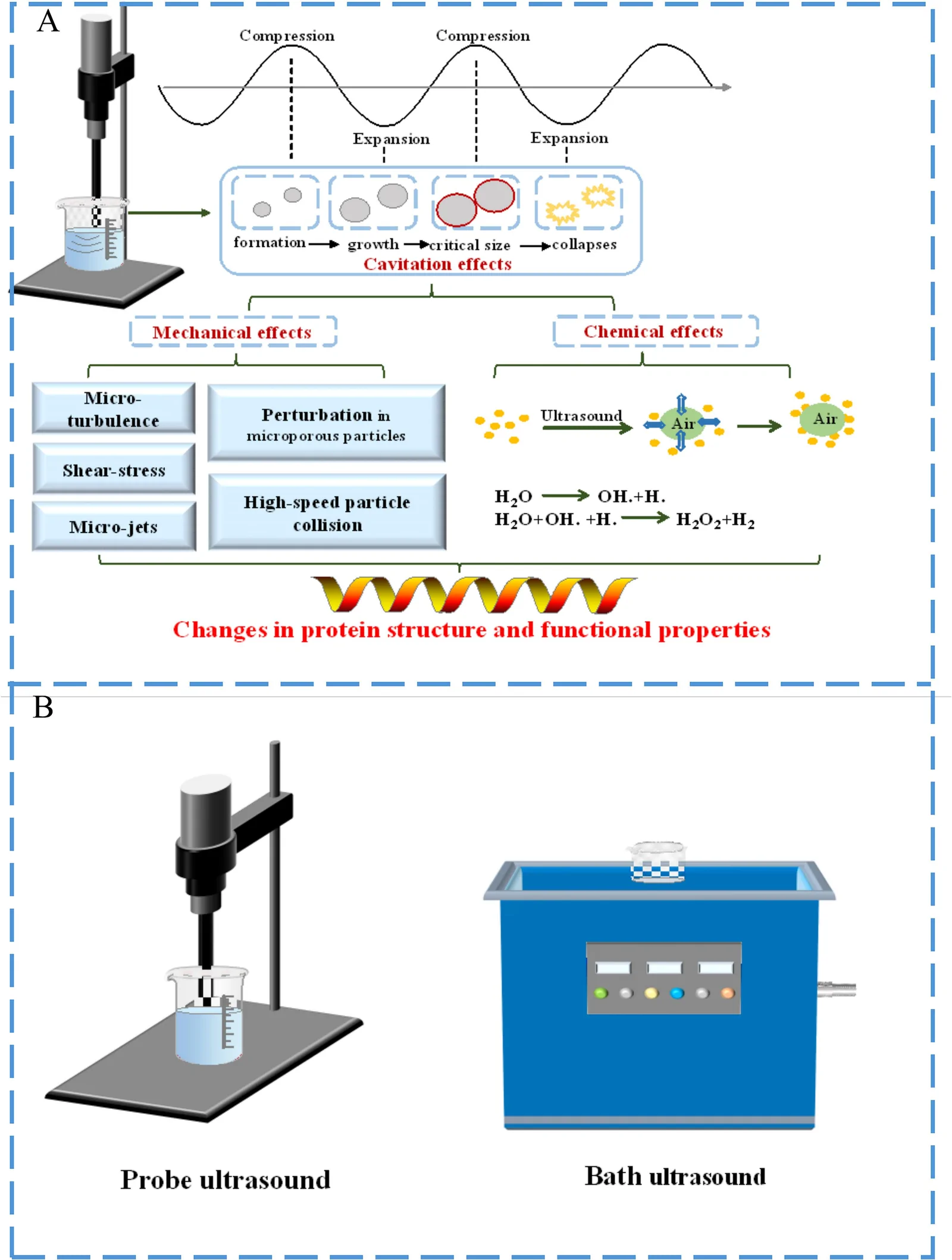
(A) Fundamental mechanisms of ultrasound action: illustrating the physical (e.g., shear, microjets), mechanical (e.g., turbulence), and chemical (e.g., radical generation) effects arising from acoustic cavitation. (B) Classification of common ultrasound equipment types (probe vs. bath-based systems) used in the preparation of protein-polysaccharide complex.

Ultrasound is defined as an elastic wave with a vibration frequency that surpasses the upper threshold of human auditory perception, typically ranging from 16 kHz to 10 MHz. The utilization of ultrasound in food science is categorized into low-intensity ultrasound and high-intensity ultrasound based on energy intensity [24]. While low-intensity ultrasound is mainly used for non-destructive quality assessment, high-intensity ultrasound (20–100 kHz, 10–1000 W/cm^2^) is particularly effective in modifying biopolymer interactions due to its strong cavitation effects (Fig. 2A) [19]. The fundamental mechanism underlying ultrasound's impact on protein-polysaccharide systems is acoustic cavitation, resulting from the interaction of acoustic waves with bubbles, which initiates most sonochemical reactions in liquid systems [25]. The ultrasound interacts with liquid molecules by longitudinal waves, causing the liquid particles to experience alternating dense and sparse states. When the tensile strain on the liquid particles surpasses the static pressure, a vacuum cavity is created, known as a cavitation bubble. Within 0.1 μs, these cavitation bubbles consistently expand to a threshold size, subsequently collapsing to generate a sequence of dynamic phenomena, including hydrodynamic shear stress, turbulence, elevated temperature, and high pressure [26]. These extreme conditions induce structural changes in proteins and polysaccharides, such as unfolding, depolymerization, and exposure of functional groups, which subsequently promote both covalent and non-covalent interactions between them.

Ultrasound cavitation is typically categorized into steady-state cavitation and instantaneous cavitation based on sound intensity. Cavitation effects produced by ultrasound with an intensity below 10 W/cm^2^ are classified as steady-state cavitation, while those above 10 W/cm^2^ are deemed transient cavitation [27]. Transient cavitation is characterized by rapid bubble collapse. It generates localized temperatures of 2000–5000 K and pressures of 300–1200 bar, which significantly enhance molecular mobility and interaction kinetics between biopolymers [14], [28]. The power and frequency of ultrasound have a direct impact on the lifespan of cavitation bubbles. Increasing the ultrasound power may lead to a larger ratio of the maximum radius of the cavitation bubble to the initial radius, thereby producing a pronounced cavitation effect. In comparison to low-frequency ultrasound, high-frequency ultrasound exhibits a shorter compression and sparse period in liquids, along with an increased propagation speed. This results in a reduced cavitation bubble volume, facilitating a more uniform yet lower intensity sound field [29]. Considering the characteristics of ultrasound cavitation bubbles, selecting appropriate ultrasonic parameters is crucial for achieving desired structural modifications in protein-polysaccharide systems.

Furthermore, the mechanical effects produced by ultrasound encompass micro-turbulence, micro-jet, high shear stress, high-speed particle collision, and disturbances in microporous particles, primarily resulting from the 110 m/s micro-jet generated by the burst of ultrasound cavitation bubbles [29]. This mechanical effect effectively overcomes the limitations associated with laminar boundary layers and enhances reaction efficiency by optimizing the transfer process between interfaces in food processing. For example, Ding et al. investigated the impact of ultrasound on the extraction efficiency and structural characteristics of soybean protein isolate, concluding that the enhanced extraction efficiency was primarily attributed to ultrasound-induced cavitation and mechanical effects. Similarly, these mechanical forces can disrupt the native structures of proteins and polysaccharides, facilitating their interaction and complex formation [30]. Moreover, the propagation of ultrasound induces thermal effects and leads to the continuous absorption of heat by the medium, resulting in a noticeable increase in temperature [31]. An appropriate increase in temperature facilitates a faster reaction rate, optimizes local nutrient distribution, and boosts enzyme activity, among other benefits. The chemical effects of ultrasound play a significant role in food processing, complementing the aforementioned impacts. The chemical effects of ultrasound primarily involve the thermal decomposition of material molecules in the medium due to high-temperature cavitation bubbles, the breakage of chemical bonds resulting from the impact of bubble explosions, and the generation of free radicals from the cracking of water molecules, with free radical chemistry being the central focus of sonochemistry. Free radicals generated by ultrasound can facilitate the interactions between different molecules, such as the covalent reaction between proteins and polyphenols as well as the Maillard reaction between proteins and polysaccharides [32]. For instance, the tea polyphenol-egg white protein conjugate was successfully prepared by ultrasound-assisted alkali/free radical method, and the results showed that ultrasound treatment improved the efficiency of TP grafting onto egg white protein and the antioxidant activity of egg white protein [36]. In another study, ultrasound treatment enhanced the Maillard reaction between sweet potato protein hydrolysates and monosaccharides, improving both reaction efficiency and product functionality [37].

### 2.2 Optimization of ultrasonic parameters for the complex formation

To achieve optimal processing results, various ultrasound working parameters such as ultrasound mode, power, frequency, working time, and amplitude are extensively researched. The majority of research has conducted single-factor experiments or orthogonal tests to refine the ultrasound parameters for optimal processing outcomes. Wang et al. investigated the impact of ultrasound treatment on peptide production from soybean meal fermented by *Bacillus amyloliquefaciens* [33]. Through single factor experiments, they identified the optimal ultrasound treatment conditions for peptide production as a synchronous dual-frequency ultrasound mode at 40/60 kHz, with a power density of 40 W/L, pulse and drop pulse durations of 40 and 60 seconds, and a total treatment time of 20 min. These parameters directly influence the extent of structural modification in proteins and polysaccharides, thereby affecting the interaction forces and functional properties of the resulting complexes.

Furthermore, in alignment with the varied methodologies for ultrasound generation, two principal types of ultrasound equipment have been developed, namely probe-based ultrasound system and bath-based ultrasound system (Fig. 2B). The choice of equipment significantly affects the efficiency of protein-polysaccharide complex formation. Probe ultrasound has extensive applications in the treatment of small volume samples, as its piezoelectric transducer can be directly immersed into the solvent. In comparison to the ultrasound bath, the ultrasound probe exhibits a more robust mass transfer effect and greater ultrasound power. This is primarily because the ultrasound probe should be directly immersed in the solution, which enhances the contact area with the material and minimizes mass transfer loss [34], [35]. This is particularly beneficial for promoting interactions between proteins and polysaccharides. Although the ultrasound apparatus of the probe presents certain inherent drawbacks, such as the significant rise of the temperature in the treated sample that could negatively impact its integrity, as well as the inconsistency in the ultrasound exposure received by the sample [36].

In addition to the aforementioned ultrasound equipment, our research group has also innovated several novel ultrasound devices, including pulsed ultrasound equipment, swept frequency ultrasound equipment, sequential multi-frequency ultrasound equipment, and synchronous multi-frequency ultrasound equipment, among others, which have been thoroughly detailed in previous reviews [27]. These advanced systems offer improved energy distribution and treatment uniformity, which are advantageous for controlling the interaction between proteins and polysaccharides. In contrast to fixed frequency ultrasound, the energy distribution of sweep frequency ultrasound exhibits greater uniformity, facilitating a more effective resonance within the material [37]. The implosion of cavitation bubbles induced by low-frequency irradiation has the potential to generate not only new cavitation nuclei for its own process but also for other ultrasound irradiations. Consequently, the implementation of multi-frequency operation may yield a greater cavitation output compared to conventional mono-frequency irradiation [38]. Generally speaking, by focusing on various food raw materials, a meticulous design of ultrasound equipment that leverages the inherent properties of ultrasound can yield more vigorous and uniform cavitation activities, presenting promising opportunities for enhancing protein-polysaccharide interactions in food production and related scientific research. In addition, when surveying the literature, it becomes evident that ultrasound power is reported using different units depending on the experimental context. Specifically, W refers to absolute power, W/L denotes power density per unit volume (relevant for scale-up), and W/cm^2^ represents power intensity per probe area (determining cavitation intensity). These units are not interchangeable and should be interpreted according to their respective contexts.

## 3 Ultrasound-assisted preparation of protein-polysaccharide complexes by changing intermolecular interactions

**Fig. 3:**
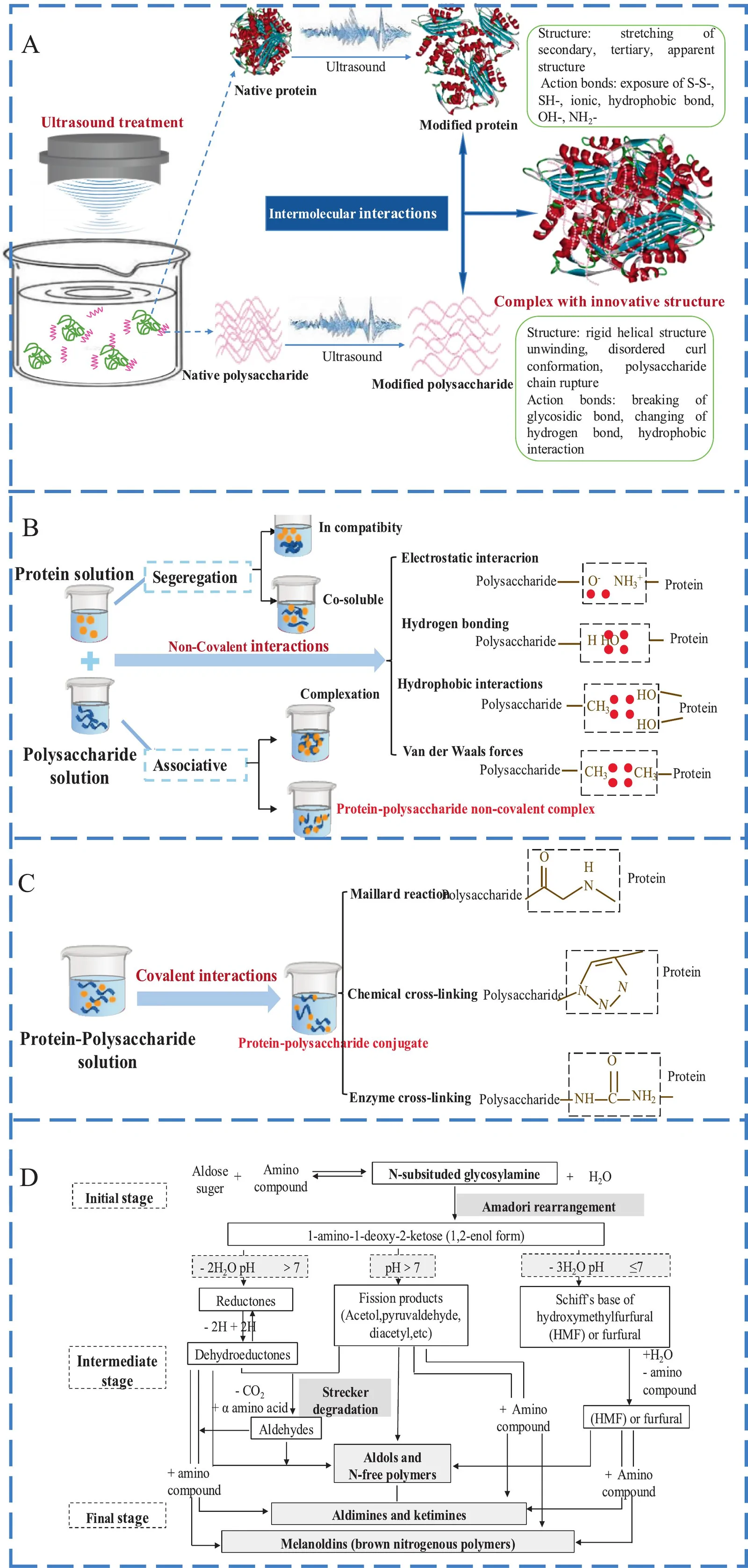
Schematic illustration of the mechanisms underlying ultrasound-assisted protein-polysaccharide complexation. (A) Proposed mechanism for the structural modification of proteins and polysaccharides induced by ultrasound. (B) Primary configurations of non-covalent complexes mediated by electrostatic, hydrophobic, hydrogen bonding, and van der Waals interactions. (C) Principal pathways for the preparation of covalent complexes, including chemical crosslinking, enzymatic crosslinking, and the Maillard reaction. (D) Detailed chemical process of the Maillard reaction between proteins and polysaccharides.

It is well known that there may be synergistic or antagonistic interactions between different types of biopolymers, resulting in great changes in the functional properties of protein-polysaccharide complexes [39], [40]. The formation of protein-polysaccharide complexes is mainly through intermolecular interactions, which can be divided into two types of intermolecular non-covalent and covalent forces. Proteins and polysaccharides are macromolecules with spatial structure. The effect of ultrasound on protein-polysaccharide composite systems essentially involved altering the structures of native macromolecular proteins and polysaccharides to modulate the exposure of interaction sites, thereby influencing their intermolecular interactions (Fig. 3A). An in-depth understanding of how ultrasound regulates the interaction between proteins and polysaccharides will be conducive to the manipulation of novel structures and physicochemical properties of protein-polysaccharide complexes in food systems. Additionally, it should be noted that ultrasound-induced structural modifications follow a biphasic pattern. Within an optimal energy window, cavitation promotes the exposure of reactive groups and enhances complexation; beyond this window, excessive shear forces can drive protein aggregation through over-exposed hydrophobic patches, while prolonged treatment may cause uncontrolled polysaccharide depolymerization. The following subsections present evidence for both the promoting effects and the critical thresholds that delimit effective processing.

### 3.1 Ultrasound-assisted preparation of non-covalent protein-polysaccharide complexes

The non-covalent interaction between proteins and polysaccharides often results in one of the configurations shown in Fig. 3B, encompassing electrostatic contacts, hydrophobic interactions, van der Waals forces, or hydrogen bonds, contingent upon the characteristics of the biopolymers and the ambient circumstances [41], [42]. These interactions are reversible and highly sensitive to molecular structure, making them particularly amenable to modulation by external physical forces. When ultrasound-induced cavitation generates localized shear forces and micro-jets, the resulting conformational changes in proteins and polysaccharides can expose previously buried interaction sites, thereby altering the balance of non-covalent forces that drive complexation [43], [44]. Understanding how ultrasound regulates these specific interaction types is therefore essential for the rational design of protein-polysaccharide complexes with tailored functional properties.

In recent years, ultrasound has been increasingly utilized to modulate the intermolecular forces between proteins and polysaccharides. Two main strategies have been explored. One strategy involves applying ultrasound to individual biopolymers prior to complexation. For example, Ji et al. employed ultrasonic pretreatment on rice protein prior to complexation with rice starch, finding that ultrasound enhanced hydrogen bonding between the two molecules and promoted gel formation through molecular entanglements and spatial confinement [45]. Similarly, ultrasound-assisted phosphorylation of cantaloupe seed protein isolate enhanced its electrostatic interaction with chitosan (–NH_3_^+^/–COO^−^), enabling stable Pickering emulsions [46]. The second, more commonly reported strategy involves ultrasound treatment of protein-polysaccharide mixtures. Kang et al. treated pullulan/oat protein film-forming solutions with ultrasound and found that it disrupted the macromolecular structures of both biopolymers, significantly enhancing intermolecular hydrogen bonding and promoting the rearrangement of macromolecular substances into a compact film network [47]. Extending this approach to more complex systems, ultrasound was applied to pre-emulsions containing SPI and sodium alginate (SA) prior to homogenization [48]. The results showed that ultrasound induced a denser and more uniform network structure in the composite gels, accompanied by increased water proton density. These improvements were attributed to an increase in β-sheet content, a decrease in α-helix content, and the enhancement of hydrogen bonding and disulfide bonding in the ultrasonicated emulsions. Interestingly, ionic bonds and hydrophobic interactions were notably weak in this emulsion system. The authors rationalized that ultrasound exposure led to the exposure of more positively charged groups on the SPI surface, which neutralized negatively charged groups and thus reduced the number of active sites available for ionic bonding with the added pork myofibrillar protein. Meanwhile, the weakened hydrophobic interactions were explained by the encapsulation of diacylglycerol hydrophobic chains by SPI or SA, which limited their contact with the hydrophobic regions of myofibrillar protein in the emulsion gel. These observations suggest that in multi-component systems, the effects of ultrasound on intermolecular interactions are not limited to the protein-polysaccharide pair but can also be modulated by other constituents. Collectively, the studies discussed above demonstrate that ultrasound can enhance non-covalent interactions including hydrogen bonding, electrostatic forces, and disulfide bonding by inducing conformational changes in proteins and polysaccharides.

Despite such evidence, a critical methodological limitation persists across most investigations. The vast majority of studies have treated the protein-polysaccharide system as an integrated whole, with the primary research objective being the improvement of functional properties rather than a mechanistic understanding of how ultrasound modulates intermolecular forces. Consequently, the differential effects of ultrasound on proteins versus polysaccharides, and how these distinct structural responses quantitatively translate into altered interaction forces, remain poorly understood [49], [50], [51]. A notable exception can be found in the work by Sun et al. who systematically examined the independent effects of ultrasound on peanut protein isolate and high–methoxyl pectin [52]. Their findings revealed a critical duality. Moderate ultrasound exposure stretched and loosened the protein structure, exposing tryptophan residues and enhancing interfacial adsorption, while also promoting hydrogen bond formation among polysaccharide molecules to form a stabilizing network. However, excessive ultrasound (8-10 min) induced protein aggregation through over‑exposure of hydrophobic groups, compromising emulsion stability. This dual‑effect nature, enhancing interactions within an optimal energy window while inducing aggregation beyond a critical threshold, underscores the need for precise parameter control and for distinguishing the individual responses of each biopolymer. Moving forward, establishing quantitative correlations between specific ultrasonic parameters and the resulting non-covalent interaction forces will be essential for the rational design of complexation processes.

### 3.2 Ultrasound-assisted preparation of covalent protein-polysaccharide

Covalent protein-polysaccharide conjugates (hereinafter referred to as CPP) are primarily prepared through the Maillard reaction, enzymatic crosslinking, or chemical crosslinking (Fig. 3C). The Maillard reaction, which is the most extensively studied approach, involves a condensation between amino groups of proteins (particularly lysine residues) and carbonyl groups of reducing polysaccharides, forming N-glucosamine followed by Amadori rearrangement to yield stable conjugates (Fig. 3D) [63]. This reaction is particularly attractive for food applications because polysaccharides' low reducibility and high steric hindrance tend to limit the reaction to its initial stage, thereby preventing the formation of colored or toxic compounds while preserving protein functionality [64]. Enzymatic crosslinking using transglutaminase or Laccase offers an alternative route with high specificity and safety [65]. In both cases, the key challenge lies in controlling reaction efficiency and uniformity, an area where ultrasound has demonstrated significant potential.

**Fig. 4:**
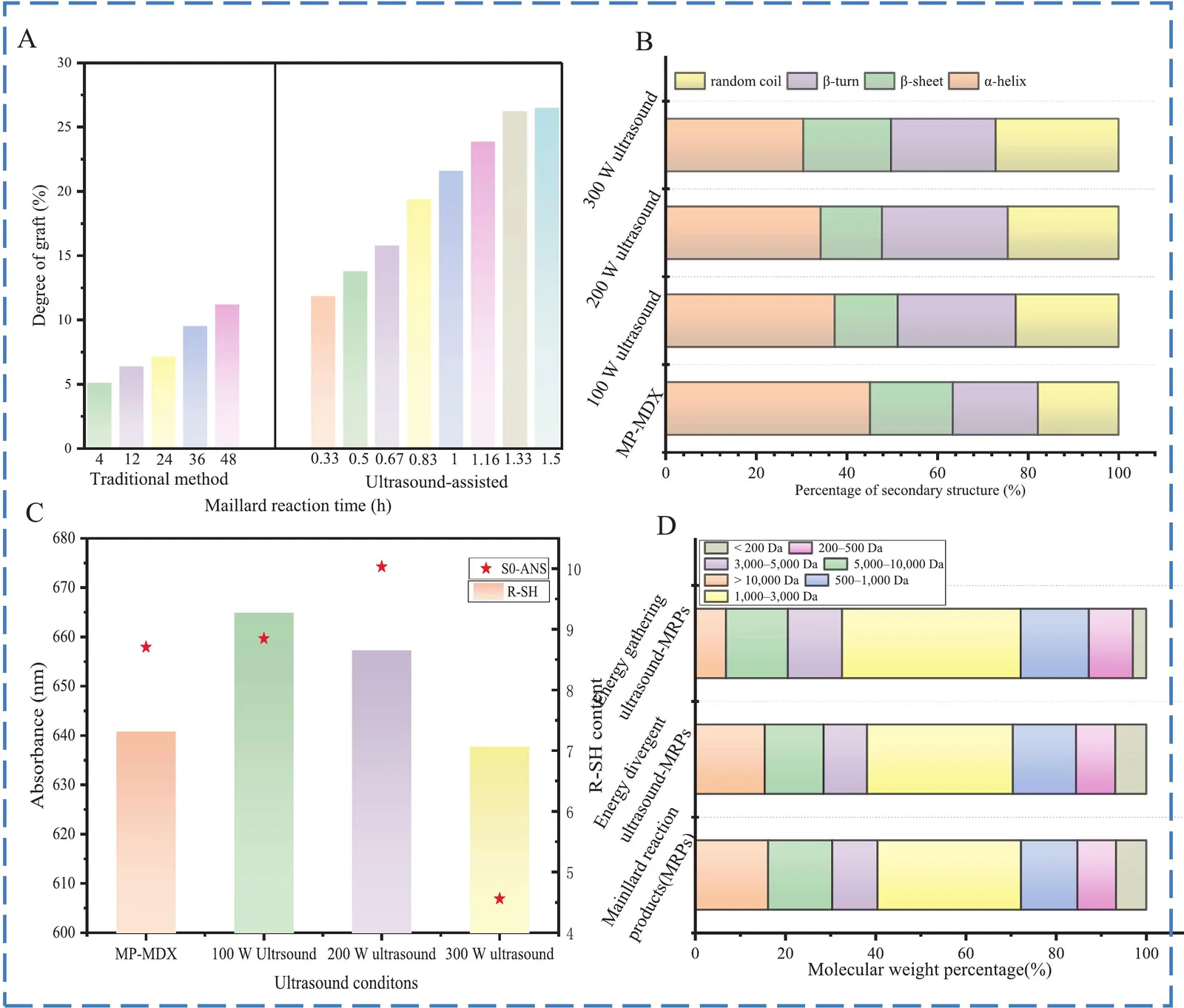
Effect of ultrasound on the properties of the protein-polysaccharide complexes. Grafting degree (A) [54]; secondary structure composition (B) and fluorescence absorption and SH content (C) [67]; molecular weight percentage (D) [68].

Over the last ten years, the synthesis of CPP through ultrasound-assisted Maillard reaction has garnered increasing interest within the food industry. The pertinent research is presented in Table 2, which indicates that the protein-polysaccharide conjugates synthesized through ultrasound-assisted Maillard reaction exhibit superior performance compared to the products derived from conventional methods. Three notable effects of ultrasound on the Maillard reaction have been documented. First, ultrasound significantly accelerates reaction kinetics. Chen et al. reported that a grafting degree of 11.20% between whey protein isolate and gum Arabic was achieved in just 20 min under ultrasound, compared to 48 h required for conventional heating [54] (Fig. 4A). Similar acceleration has been observed in other systems, including hemp protein- carboxymethyl chitosan conjugates [66] (2 h vs. 12 h) and ovalbumin-xylose systems [53]. Second, ultrasound substantially alters the structural and physicochemical properties of the resulting conjugates. At the molecular level, ultrasound treatment at 100–200 W decreases α-helix and β-sheet contents while increasing β-turn and random coil contents, indicating that cavitation disrupts the hydrogen bonding networks stabilizing ordered structures [67] (Fig. 4B-C). At the microstructural level, ultrasound produces denser and more porous networks compared to conventional heating. Additionally, ultrasound alters the molecular weight distribution of Maillard reaction products, leading to a higher proportion of lower molecular weight fractions) [68] (Fig. 4D). These structural modifications are accompanied by increased surface hydrophobicity and reactive sulfhydryl group co ntents, reflecting enhanced surface properties of the conjugates [67]. These effects are attributed to the cavitation-induced physical, mechanical, and chemical actions of ultrasound. The physical effects of cavitation, particularly shear forces and micro-jets, disrupt the tertiary structure of proteins, exposing lysine residues and other reactive amino groups that are originally buried within the protein interior [69]. These exposed groups become more accessible to reducing polysaccharides, thereby enhancing the probability of successful conjugation. The mechanical effects improve mass transfer and molecular collisions between proteins and sugars, further accelerating reaction kinetics [54]. Meanwhile, the chemical effects, particularly free radical generation from water sonolysis, may further promote conjugation efficiency [32]. Collectively, these synergistic mechanisms enable ultrasound to overcome the kinetic limitations of conventional Maillard reaction while simultaneously modifying the structural and physicochemical properties of the resulting conjugates.

**Table 2:** The protein-polysaccharide conjugates prepared by ultrasound-assisted Maillard reaction showed better properties compared to the products obtained by the traditional method.

Biopolymer Sources	Conventional method	Ultrasound conditions	Results	References
Protein: Ovalbumin (OVA) Polysaccharides: Xylose (XY)	1 mg/mL of OVA, 3:1 of XY/OVA, pH 7.0, 50 ℃	20-25 kHz, 189.5 W, 61.2 min	Glycosylation degree (DG)↑, foaming ↑, emulsifying ↑	[53]
Protein: watermelon seed protein Polysaccharides: glucose (GL)	10 mg/mL of WPI, WPI-GA ratio 1:1, pH 10, 90 °C	25 kHz, 100-300 W, 10-60 min	Solubility ↑, foaming properties ↑, emulsifying properties ↑, thermal stability ↑, antioxidant activities ↑	[54]
Protein: WPI Polysaccharides: Gellan gum (GG)	0.5-1.5 mg/mL of WPI, WPI-GG ratio 1:2, pH 10, 70 °C	100-500 W, 20-80 min	DG ↑, the emulsifying ↑, foaming ↑, solubility ↑	[55]
Protein: Pea protein isolate (PPI) Polysaccharides: GL	10 mg/mL of WPI, PPI-GL ratio 5:1, pH 10, 80°C, 0, 6, 12, 18, and 24 h	150-450 W, 5 min	Solubility↑, hydrophobicity↑, DG↑, foaming↑, emulsification ↑	[56]
Protein: Mung bean protein isolates (MBPI) Polysaccharides: GL	10 mg/mL of MBPI, MBPI-GL ratio 1:1, pH 7.8, 24 h and 80°C	20 kHz, 100-450 W, 10-20 min	Solubility ↑, emulsifying activity ↑, emulsion stability ↑, surface hydrophobicity ↑	[57]
Protein: Goat whey protein (GWP) Polysaccharides: GA	20 mg/mL of MBPI, MBPI-GL ratio 4:3, pH 7, 80°C	20-25 kHz, 200-600 W, 10-50 min	DG↑, solubility↑, emulsification↑, foaming↑	[58]
Protein: Smooth hound viscera protein (SHV) hydrolysates Polysaccharides: Sucrose (SU)	20 mg/mL of SHV, SHV-SU ratio 1:1, 2 h and 90°C	25 kHz, 160 W /cm2, 30 min, 40℃	DG ↑, antioxidant activity ↑	[59]
Protein: Chinese giant salamander skin collagen (CGSS) Polysaccharides: G /XY	10 mg/mL of CGSS, CGSS-GL-XY ratio 0.3:7.5:7.5, 1 h and 80°C	600 W, 1 h, 80°C	DG ↑, surface hydrophobicity ↑, antioxidant activity ↑	[60]
Protein: Mussel meat protein hydrolysate (MMP) Polysaccharides: G /XY	30 mg/mL of MMP, MMP-GL ratio 97:2:1, 2 h and 115°C, pH 6	25 kHz, 300 W, 2 h	Antioxidant activity ↑	[61]
Protein: Chicken liver protein (CLP) Polysaccharides: XY	50 mg/mL of CLP, CLP-XY ratio 1:1, 1.5 h and 120°C, pH 6	200 W, 12 min	The utilization rate of amino acids↑	[62]

Note: “↑” indicates that the conjugate prepared by ultrasound-assisted Maillard reaction exhibits significantly improved properties compared with the sample prepared by conventional heating alone. Power units are reported as in the original references.

In addition to the Maillard reaction, enzymatic glycosylation via transglutaminase offers an alternative route for preparing protein-polysaccharide covalent conjugates. TGase catalyzes the acyl transfer reaction between glutamine residues in proteins and primary amino groups of aminated polysaccharides, forming stable protein-saccharide conjugates [52]. However, the efficiency of this reaction is often constrained by the native folded structure of proteins, which buries glutamine residues and limits their accessibility to the enzyme. Ultrasound overcomes this limitation by inducing protein unfolding that exposes more glutamine residues for TGase catalysis. For example, ultrasound pretreatment of sodium caseinate at 400 W for 10 min significantly increased the grafting degree with carboxymethyl chitosan. Structural analyses revealed increased surface hydrophobicity, elevated fluorescence intensity, and reduced particle size, indicating that cavitation-induced unfolding exposed more reactive sites for conjugation [70]. These findings demonstrate that ultrasound enhances TGase-mediated protein-polysaccharide conjugation through protein unfolding and improved enzyme-substrate interactions, offering a mild and specific alternative to the Maillard reaction. However, because TGase requires aminated polysaccharides as acyl acceptors, whereas most food-grade polysaccharides lack primary amino groups, the application of this approach in food systems remains relatively limited. Nevertheless, the promoting effect of ultrasound on TGase catalysis has been well documented in protein crosslinking systems, providing valuable mechanistic insights [71]. Wang et al. found that ultrasound pretreatment reduced the gel-point temperature of pea protein from 44 °C to 28°C during TGase-catalyzed crosslinking, though extending ultrasound to 20 min reversed this benefit, indicating an optimal pretreatment window [72]. Ultrasound also enhances laccase-mediated crosslinking by disrupting protein structure and exposing tyrosine residues and hydrophobic groups, the primary sites for laccase catalysis, thereby improving subsequent crosslinking efficiency [73]. Collectively, these examples reveal a common mechanistic theme. Regardless of the enzyme type (TGase or laccase) or the crosslinking target, ultrasound serves as a structural activator that enhances enzymatic crosslinking efficiency through protein unfolding and exposure of reactive sites. This stands in contrast to the Maillard reaction, where ultrasound not only promotes protein unfolding but also directly contributes through free radical-mediated pathways and accelerated mass transfer. In enzymatic systems, the role of ultrasound is primarily indirect, creating favorable conformational conditions for enzyme catalysis rather than directly participating in the reaction chemistry. This distinction suggests that ultrasound may be more broadly applicable as a pretreatment strategy for enzyme-mediated protein modifications, where substrate accessibility is a limiting factor.

## 4 Techno-functional properties of ultrasound-assisted protein-polysaccharide complexes

It is widely recognized that protein-polysaccharide complexes are extensively utilized in the food industry. These substances serve as alternatives to fat, acting as emulsifiers, thickeners, or stabilizers, thereby improving the texture and rheological properties of food products [40], [74]. The advancement of protein-polysaccharide complexes in functional foods holds considerable importance, as it can contribute to the structuring of these foods and enhance their functional properties.

### 4.1 Solubility enhancement through ultrasound-assisted complexation

**Fig. 5:**
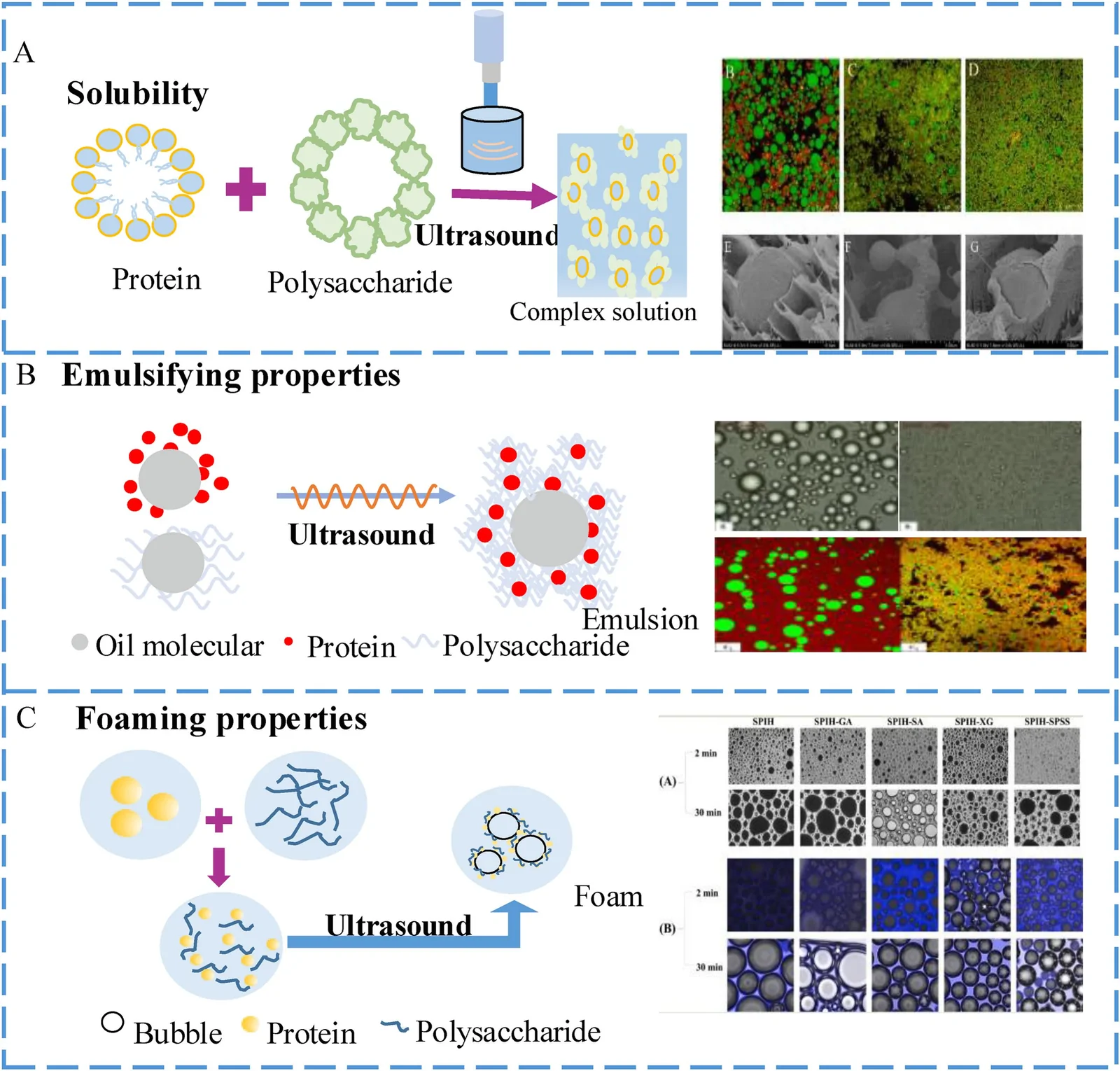
Potential mechanisms of ultrasound to enhance the solubility (A), emulsifying properties (B) and foaming properties (C) of protein-polysaccharide complexes. The microscopic morphologies of the above three diagrams are from Chen. et al., [76], Zhou et al. [123] and Zhang et al. [124], respectively.

Solubility, a fundamental characteristic of protein, is intricately linked to its functional qualities, including emulsification and dispersion [75]. Ultrasound-assisted glycosylation has been developed as an efficient strategy for generating highly soluble protein-polysaccharide conjugates. For example, the solubility of ovalbumin after ultrasound-assisted glycosylation progressively improved with extended reaction time, reaching maximum at 120 min [76]. Similarly, ultrasound-assisted glycosylation of rice protein with dextran significantly increased its solubility, attributed to increased grafting degree and reduced arginine and lysine content [77]. These improvements result from the covalent attachment of hydrophilic polysaccharide chains, which enhances the overall hydrophilicity of the conjugates, and from ultrasound-induced structural modifications that expose more charged and polar residues on the protein surface. The efficacy of this approach is also influenced by the molecular weight and chain conformation of the polysaccharide, as bulky polysaccharides may hinder effective conjugation if not properly aligned through ultrasonic mixing. This notion is further supported by the work of Wang et al., who demonstrated that ultrasound-assisted covalent conjugation of peanut protein isolate with high-methoxyl pectin significantly enhanced solubility across a broad pH range. At pH 5.0, near the isoelectric point where native protein aggregates sharply, the conjugates exhibited only a slight decrease in solubility, attributed to the spatial site-blocking effect of the grafted polysaccharide chains that inhibited aggregation through steric hindrance (Fig. 5A) [78].

Beyond covalent conjugation, ultrasound-assisted non-covalent complexes also enhance protein solubility. Notably, the sequence of ultrasound and polysaccharide addition critically influences the outcome. When yeast protein was complexed with low-methoxyl pectin followed by ultrasound, the complexes exhibited the highest dispersible protein fraction, whereas the reverse sequence was less effective, indicating that the presence of polysaccharide during ultrasound exposure is essential for capturing unfolded protein intermediates and preventing their aggregation [79]. Similarly, ultrasound-assisted complexation of soybean protein isolate with inulin at an optimal concentration of 20 mg/mL enhanced solubility to 55.51%, accompanied by reduced particle size and increased ζ-potential. However, excessive inulin led to a decrease in solubility due to competition for water molecules, revealing a polysaccharide concentration threshold beyond which the benefits are reversed [80]. Collectively, these results demonstrate that the presence of polysaccharides during ultrasound exposure, whether through covalent conjugation or non-covalent complexation, can effectively capture ultrasound-unfolded protein intermediates and enhance solubility across a broader pH range. However, the effectiveness of this approach is highly system-dependent, requiring case-by-case optimization of ultrasonic parameters, polysaccharide selection, addition sequence, and biopolymer ratio. This system-specific nature highlights the importance of moving beyond empirical screening toward mechanistic principles that can guide the design of each system's ultrasound response.

### 4.2 Emulsifying properties tailored by ultrasound treatment

Emulsions stabilized by protein-polysaccharide complexes have garnered significant interest due to their superior stability compared to systems stabilized by individual biopolymers [81]. The synthesized amphiphilic protein-polysaccharide complexes can be securely affixed at the oil-water interface via the protein's hydrophobic region, forming a viscoelastic layer, while the polysaccharide region offers substantial steric hindrance to inhibit flocculation and coalescence (Fig. 5B) [82]. Compared to conventional methods, ultrasound treatment markedly improves the emulsifying properties of protein-polysaccharide complexes. Two prominent indices that characterize the emulsifying properties of protein-polysaccharide complexes are the emulsifying activity index (EAI) and the emulsion stability index (ESI) [83]. The former illustrates the protein's capacity to adsorb at the interface, whilst the latter demonstrates how the protein imparts strength to the emulsion, enabling it to endure structural alterations over a certain duration. For instance, ultrasound-assisted glycosylation of rapeseed protein isolate with dextran significantly improved its emulsifying capacity, resulting from ultrasound-induced protein unfolding that exposed hydrophobic groups and promoted interfacial adsorption [84]. Similarly, ultrasound-assisted heating significantly accelerated the Maillard reaction between brewer's spent grain protein and gum Arabic, achieving a 115% increase in emulsifying activity index and a 13% increase in emulsion stability index in just 45 min. In contrast, conventional heating required 3 h to attain comparable emulsifying activity [85]. Structural analysis revealed that ultrasound increased α-helix content, which is favorable for oil-water interface stabilization, whereas conventional heating enhanced flexibility through increased random coil content, both pathways leading to improved emulsifying performance. In fact, for covalent conjugates prepared via ultrasound-assisted Maillard reaction, the enhanced EAI and ESI are attributed to increased surface hydrophobicity, improved molecular flexibility, and reduced particle size, which collectively facilitate rapid adsorption and rearrangement at the oil-water interface. Beyond covalent conjugation, ultrasound-assisted non-covalent complexation also improves emulsifying properties [82], [86]. For example, when yeast protein was complexed with low-methoxyl pectin followed by ultrasound, interfacial protein adsorption increased from 20.5% to 68.7% and surface excess from 1.05 to 3.35 mg m^−2^, which translated into enhanced EAI and ESI [79]. Multi-frequency power ultrasound was used to modulate the complexation of sodium caseinate and pectin to enhance the emulsifying characteristics of the resultant complex. The findings indicated that the EAI and ESI of the caseinate-pectin complex were elevated by 33.12% and 7.27%, respectively, after ultrasound treatment at a frequency of 60 kHz, a power density of 50 W/L, and a duration of 25 min [87]. Notably, these improvements are not unlimited. Ma et al. systematically investigated the emulsifying properties of soy protein isolate-citrus pectin complexes under different ultrasonic powers and found that both emulsifying activity and stability reached maximum values at 630 W, but declined at 720 W [88]. This non-linear behavior echoes the threshold phenomenon observed in solubility enhancement (Section 4.1) and in non-covalent/covalent systems (3.1, 3.2), further supporting the notion that an optimal energy threshold may be a universal principle governing ultrasound-assisted protein-polysaccharide interactions. The mechanisms underlying this threshold behavior are twofold. Moderate ultrasound promotes protein unfolding and enhances interfacial adsorption, while excessive energy induces protein aggregation and reduces interfacial adsorption capacity [76]. Identifying the specific ultrasonic parameters that yield optimal emulsifying properties for each system remains a priority for mechanism-guided optimization.

### 4.3 Foaming characteristics improved via ultrasound modification

Foaming properties, including foaming capacity (FC) and foam stability (FS), are critical for protein-polysaccharide complexes in applications such as aerated food products [89]. The ability of proteins to adsorb rapidly at the air-water interface and form a cohesive film determines foam performance, which can be enhanced through polysaccharide incorporation and ultrasound treatment (Fig. 5C). For covalent conjugates prepared via ultrasound-assisted Maillard reaction, enhanced foaming properties have been widely documented. For example, ultrasound-assisted glycosylation of brewer's spent grain protein with maltose enhanced FC from 82.22% to 165.10% and FS from 10.60% to 131.20%, with the treated conjugates exhibiting a lower foam collapse rate compared to ultrasound or glycosylation alone [90]. These improvements are attributed to increased surface hydrophobicity, reduced particle size, and enhanced molecular flexibility, which facilitate rapid adsorption and film formation at the air-water interface [91], [92]. For non-covalent complexes, the combination of ultrasound and polysaccharide complexation also enhances foaming properties through synergistic effects. Notably, the processing sequence critically influences the outcome. When yeast protein was complexed with low-methoxyl pectin followed by ultrasound, the complexes exhibited the highest FC and FS, whereas ultrasound alone or the reverse sequence produced only intermediate improvements [79]. This superior performance was attributed to backbone reorganization (helix/turn→β-sheet) and the formation of a cohesive protein-pectin corona that resisted lamella drainage and bubble coalescence [70]. This trend mirrors the emulsifying properties observed for the same system (Section 4.2), suggesting that the synergistic mechanism applies broadly to both oil-water and air-water interfaces.The molecular weight of polysaccharides further modulates foaming properties in ultrasound-assisted systems, with low-molecular-weight chitosan inducing greater protein unfolding and exposing more hydrophobic groups and disulfide bonds, thereby enhancing foaming ability and foam stability [93]. Moreover, the ultrasound-polysaccharide synergistic mechanism can be amplified by integrating additional processing technologies, as demonstrated by microwave-ultrasound-pectin treatment of liquid egg white, which increased FC by 24.64% and FS by nearly 230% compared to ultrasound alone [94].

Together, these findings reveal a complementary mechanism distinct from the energy-dependent threshold behavior governing solubility and emulsifying properties (4.1, 4.2). Foaming properties are shaped by a qualitative division of labor, where ultrasound enhances foaming capacity and polysaccharides ensure foam stability. This synergy can be further amplified by integrating additional processing technologies, such as microwave. The practical implication is that optimizing foaming properties requires not only precise ultrasonic parameter control but also careful selection of polysaccharide type, molecular weight, and addition sequence to achieve synergistic enhancement of both foaming capacity and stability.

## 5 Application of sonicated protein-polysaccharide complexes in the food industry

**Fig. 6:**
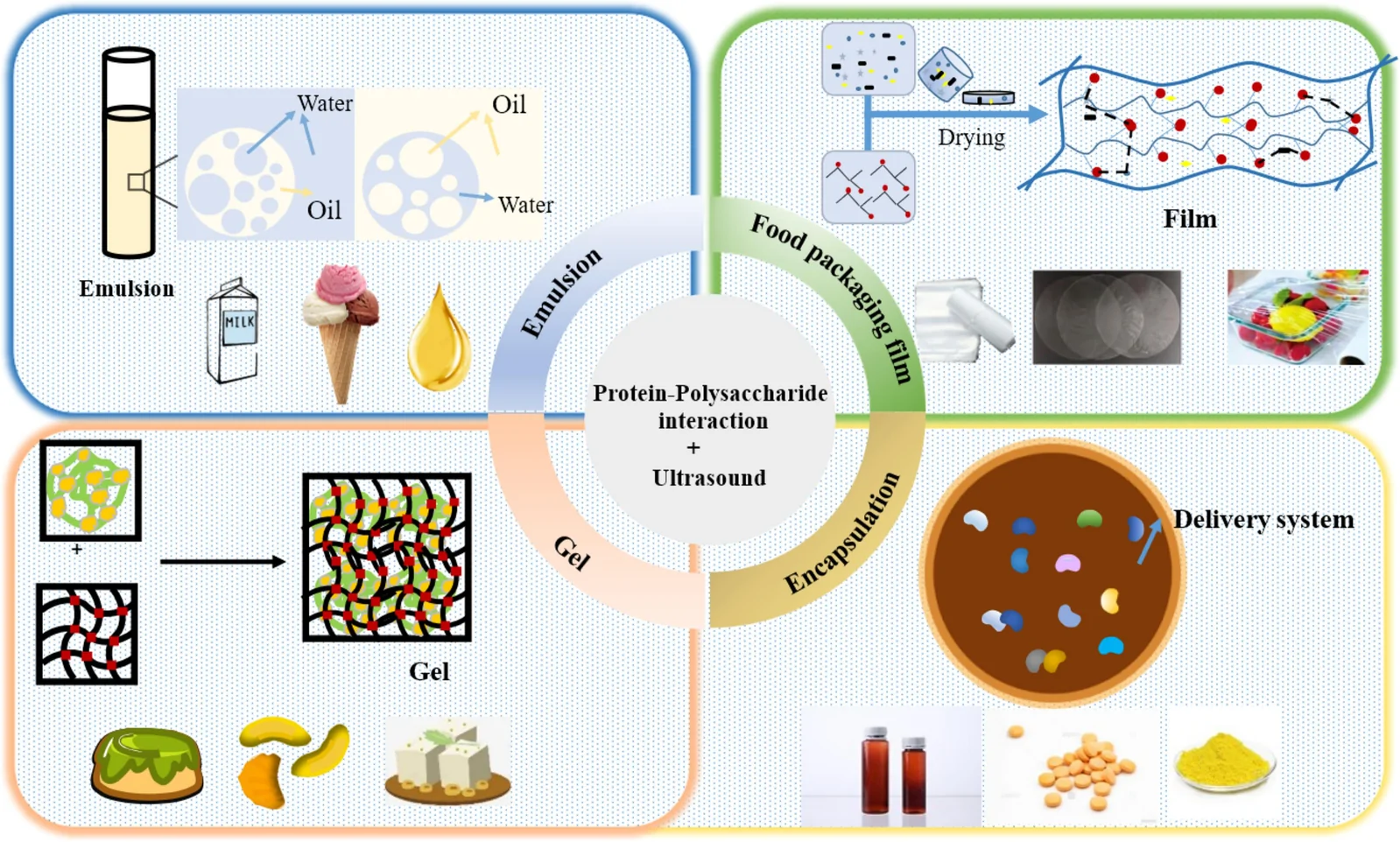
A specific application regarding ultrasound-assisted preparation of protein-polysaccharide complexes.

The enhanced functional properties of ultrasound-assisted protein-polysaccharide complexes, including improved solubility, emulsification, and foaming, have motivated their exploration in various food applications. The mechanistic insights established in 3, 4, particularly ultrasound-induced protein unfolding, exposure of reactive groups, and the synergistic role of polysaccharides, provide a foundation for understanding how these complexes perform in real food systems. This section discusses the applications of ultrasound-assisted protein-polysaccharide complexes in emulsions, gels, bioactive compound delivery, and edible packaging films, with an emphasis on the structure-function relationships that govern their performance (Fig. 6). Nevertheless, while these applications demonstrate considerable promise, their practical translation is constrained by several factors, including batch-to-batch variability in cavitation fields, energy efficiency at industrial scale, and the potential for ultrasound-induced oxidation or sensory changes in complex food matrices. The application performance discussed here directly translates the mechanistic modifications (Chapter 3) and functional enhancements (Chapter 4) established above into real food systems.

### 5.1 Ultrasound-stabilized emulsions using protein-polysaccharide complexes

As discussed in Sections 4.2 and rooted in the mechanistic principles of Section 3.1, ultrasound enhances the emulsifying properties of protein-polysaccharide complexes by inducing protein unfolding, exposing hydrophobic groups, and promoting interfacial adsorption. These structural modifications translate directly into improved emulsion stability in practical applications. Ultrasound can be applied at two different stages of emulsion preparation. The first strategy involves pre-treating protein-polysaccharide complexes with ultrasound before emulsification. For example, ultrasound treatment of casein-hyaluronic acid complexes at 150 W for 10 min reduced particle size, enhanced electrostatic repulsion, and altered intermolecular interactions, enabling the formation of emulsions with improved creaming stability [95]. Similarly, ultrasound treatment of pea protein-high methoxyl pectin complexes at pH 2.0 promoted their self-assembly into core-shell structured particles with increased α-helix and β-sheet contents, which subsequently served as effective emulsion stabilizers [96]. The second strategy involves applying ultrasound directly to the protein-polysaccharide-oil mixture during emulsification, enabling simultaneous complexation and emulsion formation. For instance, dual-frequency countercurrent probe ultrasound (DFU, 20/28 kHz) applied to sodium caseinate-pectin-stabilized emulsions significantly reduced droplet size from 12.60 to 3.09 μm and increased the stability index to 97%, outperforming high-speed shear homogenization [4]. Interfacial adsorption of both casein (from 3.55 to 6.29 mg/m^2^) and pectin (from 3.28 to 5.83 mg/m^2^) was substantially enhanced under DFU treatment. Similarly, ultrasound-assisted homogenization of SPI-pectin mixtures decreased interfacial tension and improved the creaming index of the resulting emulsions [97], while ultrasound treatment of fish myofibrillar protein-xanthan gum mixtures improved emulsion stability with reduced droplet size and increased ζ-potential [98]. This one-step approach offers advantages for industrial applications where process efficiency is prioritized. The two strategies differ not only in processing workflow but also in the depth of mechanistic understanding they afford. Pre-treating complexes before emulsification provides a clearer mechanistic picture, as the structural changes induced by ultrasound (e.g., particle size, surface hydrophobicity, secondary structure) can be characterized independently of the oil phase, enabling a more direct correlation between complex structure and emulsion performance. In contrast, applying ultrasound directly to the emulsion mixture introduces simultaneous effects on oil droplet breakup, interfacial reorganization, and complex modification, making it more challenging to decouple the individual contributions of each mechanism. This complexity often requires additional control experiments, such as comparing ultrasound-treated versus untreated complexes under identical emulsification conditions, to isolate the specific role of ultrasound in emulsion stabilization.

For both strategies, ultrasound treatment consistently reduces particle size, increases surface hydrophobicity, and alters secondary structures, which collectively enhance the adsorption capacity of complexes at the oil-water interface, as evidenced by reduced interfacial tension (e.g., 26.7 mNm^−1^) and increased interfacial protein adsorption [98]. The resulting emulsions demonstrate significantly improved stability across multiple dimensions. DFU-treated caseinate-pectin emulsions exhibited remarkable resistance to thermal stress (25–75 °C) and enhanced freeze-thaw stability, as evidenced by backscattering spectra and crystallization temperature changes [4]. Moreover, DFU treatment effectively delayed auto-oxidation during 96 h of storage, as indicated by reduced peroxide and malondialdehyde values. SPI-pectin complexes similarly exhibited enhanced storage durability with reduced creaming index compared to untreated counterparts [97], while pea protein-high methoxyl pectin complexes-based emulsions prepared by ultrasound formed small, dispersed oil drops that remained stable during storage without creaming [96]. Pickering emulsions stabilized by ultrasound-assisted phosphorylated cantaloupe seed protein-chitosan complexes also exhibited temperature- and ionic strength-dependent stability: they remained stable at 4–25 °C but stratified at 40–65 °C, while 90 °C induced gel formation; increasing NaCl concentration weakened electrostatic interactions, leading to droplet aggregation and increased creaming [46]. Notably, the enhanced emulsion stability also benefits the protection of encapsulated bioactive compounds. DFU treatment increased the encapsulation efficiency and bioavailability of quercetin, with the antioxidant activity of the encapsulated quercetin remaining stable during 24 h of storage, in contrast to the control sample where it decreased significantly [4]. These findings collectively demonstrate that ultrasound not only stabilizes the emulsion structure itself but also creates a protective environment for encapsulated bioactive compounds against environmental stressors, as further discussed in Section 5.3.

Despite these advances, practical applications of ultrasound-stabilized emulsions in real food products remain limited. Most studies have been conducted at laboratory scale, and the translation of these findings to industrial-scale continuous operations presents significant challenges, including reactor design for uniform energy distribution, energy efficiency optimization, and process scalability. Bridging this scale-up gap will be critical for commercial adoption, alongside exploring emerging areas such as Pickering emulsions stabilized by ultrasound-modified protein-polysaccharide particles.

### 5.2 Ultrasound-fabricated gels and texturized foods

Ultrasound modulates the structural conformation of macromolecules, thereby altering the balance of intermolecular forces between proteins and polysaccharides to enable the fabrication of composite gels with enhanced textural and functional properties [99], [100]. Ultrasound-assisted protein-polysaccharide gels can be broadly classified into hydrogels and emulsion gels, each with distinct structural characteristics and application scenarios.

Hydrogels, formed by the crosslinking of proteins and polysaccharides into three-dimensional polymeric networks [101], benefit from ultrasound through enhanced intermolecular interactions and improved network uniformity. In peanut protein isolate-Tremella polysaccharide composite hydrogels, ultrasonic treatment at varying powers (0–600 W) revealed a clear biphasic response: the minimum particle size (12.16 μm) and optimal gel strength were achieved at 400 W, while excessive power (600 W) induced structural disruption. CLSM and SEM confirmed that moderate ultrasound reformed the network into a more compact and homogeneous structure [102]. Similar effects were observed in corn germ globulin-Tremella polysaccharide hydrogels, where ultrasonic pretreatment at 300 W with a 1:1 protein-to-polysaccharide ratio promoted electrostatic self-assembly, forming a stable amphiphilic complex with enhanced water-holding capacity [103]. Emulsion gels, which combine emulsion droplets dispersed within a gel matrix, offer advantages for encapsulating lipophilic bioactive compounds and serving as fat replacers [104]. Ultrasound-treated soy protein-hyaluronic acid conjugates formed high internal phase Pickering emulsion gels with gel strength nearly twice that of SPI-based gels (G′: 8389.2 Pa vs 4187.9 Pa), with the dense network enabling high-precision 3D printing and compliance with the International Dysphagia Diet Standardisation Initiative (IDDSI) Level 5 criteria for dysphagia-friendly foods [105]. For emulsion gels requiring enzymatic crosslinking, ultrasound pretreatment of soybean protein isolate (200 W, 10 min) combined with TGase and 0.3% gum Arabic achieved 95.05% water-holding capacity and 84.05 N hardness, a 2.6-fold enhancement over untreated gels [106]. For fat-replacer applications, silkworm pupa protein-carrageenan Pickering emulsions incorporated into pork myofibrillar protein gels enhanced hardness by 18-fold and achieved 83.6% vitamin A encapsulation efficiency [107].

Beyond conventional gelation, ultrasound-assisted protein-polysaccharide gels have found emerging applications in several advanced areas. In 3D food printing, the rheological properties of gels, including shear-thinning behavior, yield stress, and rapid structural recovery, are critical for printability. Ultrasound-assisted fabrication of ovalbumin-gellan gum emulsion gels demonstrated that ultrasonication time critically modulates printing precision, with an optimal treatment time of 3.5 min achieving the highest printing accuracy (15%) [108]. This non-linear behavior mirrors the energy-dependent threshold effects discussed in 3.1, 4.2. This emulsion gels stabilized by ultrasound-treated soy protein-hyaluronic acid conjugates further confirmed this potential, achieving high printing precision (height: 7.94 mm vs 8.00 mm theoretical) with excellent shear-thinning behavior and structural recovery after extrusion [105]. In dysphagia management, protein-polysaccharide composite hydrogels have been designed as texture-modified foods that meet IDDSI requirements. Ultrasound-treated soy protein-hyaluronic acid emulsion gels successfully passed IDDSI Level 5 criteria, exhibiting soft texture, low adhesiveness, and easy deformation under mild pressure, characteristics that reduce swallowing risk.

These examples collectively demonstrate that ultrasound serves as an effective tool for tailoring gel microstructure and mechanical properties across diverse gel types and application scenarios. The extent of improvement depends on the specific biopolymer combination, gel architecture, ultrasonic conditions, and the synergistic integration of complementary processing technologies such as enzymatic crosslinking. Future research should prioritize expanding the functional applications of ultrasound-assisted composite gels, including controlled release systems for nutrient delivery, precision manufacturing through 3D food printing, and the development of dysphagia-oriented soft foods and satiety-enhancing functional foods.

### 5.3 Ultrasound-engineered edible packaging films

Edible films based on proteins and polysaccharides have emerged as sustainable alternatives to traditional plastic packaging [109]. Ultrasound enhances their performance by promoting intermolecular hydrogen bonding and crosslinking between biopolymers, thereby improving mechanical strength, barrier properties, and structural uniformity. In the most straightforward paradigm, ultrasound is applied directly to film-forming solutions to modify biopolymer structures and enhance film properties [110]. For example, ultrasound treatment of rice protein hydrolysate-chitosan films disrupted the original macromolecular structure of chitosan, exposing more active sites for hydrogen bonding with the protein, which improved oxygen barrier properties and established a compact network structure [111]. High-intensity ultrasound combined with transglutaminase enhanced the tensile strength and thermal stability of quinoa protein-chitosan films by promoting protein-polysaccharide crosslinking and forming a more ordered secondary structure [112], while ultrasound-assisted fabrication of nisin-loaded oat protein-pullulan films not only improved mechanical properties and intermolecular hydrogen bonding but also exhibited effective antibacterial activity against *Listeria monocytogenes* and *Escherichia coli*, extending the shelf life of strawberry fruits during storage [47]. Beyond this conventional role, ultrasound has demonstrated three emerging and more sophisticated functions in film fabrication beyond the direct processing of film-forming solutions. First, ultrasound can serve as an extraction tool to recover functional components from food waste. Ultrasound-microwave assisted co-extraction from red grape pomace simultaneously yielded pectin and polyphenols, which were then incorporated into kidney bean protein films, achieving tensile strength up to 45.41 MPa with antioxidant and antibacterial activity [113]. Second, ultrasound alone can effectively construct the film-forming matrix itself, thereby improving subsequent film performance. When applied during the lag phase of arachin amyloid-like fibril formation, it enhanced interfacial functionality more effectively than post-treatment, improving the performance of chitosan-based composite films [114]. Third, ultrasound can act on emulsions, allowing the film-forming matrix to be presented in the form of nanoemulsions. In a study from our group, ultrasound-assisted nanoemulsions (600 W, 10 min) of soy protein with curcumin or orange essential oil were incorporated into pullulan-based films, improving water vapor permeability, tensile strength, and antibacterial activity against *Escherichia coli* and *Staphylococcus aureus*, while effectively extending strawberry shelf life [6].

These examples illustrate that ultrasound's role in edible film fabrication has evolved from a direct processing aid to a multifunctional platform encompassing the extraction of functional components, the construction of film-forming matrices, and the preparation of nanoemulsion-based film systems [115]. Given the inherent complexity and diversity of film-forming matrices, ranging from proteins and polysaccharides to bioactive extracts and nanoemulsions, the timing of ultrasound intervention and the specific target of its action have become critical determinants of film performance. A deeper mechanistic understanding of how ultrasound interacts with different matrix components at different stages of film assembly will be key to rational process design.

### 5.4 Ultrasound-enabled delivery systems for bioactive compounds

The encapsulation of bioactive compounds within protein-polysaccharide complexes has emerged as a promising strategy to enhance their stability and bioavailability [116]. Ultrasound contributes to this application by leveraging the structural regulation mechanisms established in Section 3.1, promoting the formation of dense, uniform nanoparticles with improved encapsulation efficiency and compact carrier structures that effectively protect bioactive compounds [117]. Ultrasound-assisted protein-polysaccharide complexes have demonstrated efficacy as delivery vehicles for various bioactive compounds. For example, ultrasound-assisted pectin-chitosan complexes achieved an encapsulation efficiency of 93% for cinnamaldehyde [118]. Dual-frequency ultrasound (28/40 kHz) enhanced resveratrol encapsulation in corn gluten-chitosan complexes to 65.2% [5]. In zein-gum Arabic complexes, ultrasound treatment (20/40 kHz, 15 min) increased encapsulation efficiency from 55.85% to 74.20% [119]. In CPP-chitosan nanoparticles loaded with quercetin, multi-frequency ultrasound (20/35/50 kHz, 80 W/L, 20 min) increased encapsulation efficiency from 64.32% to 78.55% [120]. These improvements consistently demonstrate that ultrasound enhances the loading capacity of protein-polysaccharide carriers for diverse bioactive compounds.

The improved encapsulation efficiency directly contributes to enhanced bioactivity retention. During simulated gastrointestinal digestion, free nobiletin degraded rapidly with only 19.58% bioaccessibility, whereas encapsulation within HMP-PPI conjugates maintained 72.13% bioaccessibility [78]. Similarly, free quercetin lost over 90% of its activity during digestion, while CPP-chitosan nanoparticles maintained approximately 49% retention [120]. Under UV irradiation, free quercetin degraded to below 60% after 210 min, whereas ultrasound-treated CPP-chitosan nanoparticles retained 85.87%. During 28 days of storage, ultrasound-treated CPP-chitosan nanoparticles retained 66.66% of quercetin, compared to only 43.03% for CPP-Qu nanoparticles. These results confirm that ultrasound-induced structural modifications create a protective environment that preserves the biological activity of encapsulated compounds against environmental and gastrointestinal stresses. Importantly, these delivery systems also exhibit sustained release behavior due to the natural biodegradability of protein and polysaccharide wall materials. In HMP-PPI-nobiletin conjugates, only 11.18% of nobiletin was released in the gastric phase, compared to 68.49% for free nobiletin, with sustained release throughout intestinal digestion. Similarly, ultrasound-treated zein-GA-resveratrol nanoparticles demonstrated controlled release profiles, attributed to the gradual breakdown of the carrier matrix in gastrointestinal environments [119]. This sustained release ensures that bioactive compounds reach their target sites in the intestine, maximizing their physiological functions.

The formation of stable nanoparticles is driven by synergistic physical and molecular interactions. Physical forces from cavitation-induced shear and microjets facilitate particle size reduction and uniform dispersion [121], [122]. Meanwhile, molecular interactions between bioactive compounds and the carrier matrix, including hydrogen bonding, electrostatic interactions, and hydrophobic forces, enhance loading capacity and structural integrity. In zein-GA-resveratrol nanoparticles, FTIR confirmed hydrogen bonding (peak blue-shifted from 3312 to 3309 cm^−1^) and CD spectroscopy revealed increased α-helix content from 33.75% to 39.90%, indicating stronger molecular [119]. In CPP-chitosan-quercetin nanoparticles, ultrasound red-shifted hydrogen bonds (3439→3302 cm^−1^) and strengthened amide I and II bands, confirming enhanced electrostatic interactions [120]. In HMP-PPI-nobiletin conjugates, intrinsic fluorescence revealed that glycation exposed tryptophan residues to a more hydrophilic microenvironment, while surface hydrophobicity significantly decreased from 7086 to 181-5008, confirming the hydrophilic nature of the carrier surface. Structural characterization further elucidates the mechanisms underlying ultrasound-enhanced encapsulation. XRD analysis revealed that ultrasound transformed crystalline bioactive compounds into amorphous states within the carrier matrix, improving their dispersibility and bioavailability. In zein-GA-resveratrol nanoparticles, ultrasound shifted diffraction peaks and increased intensity, indicating structural rearrangement and enhanced intermolecular crosslinking [119]. In CPP-chitosan-quercetin nanoparticles, ultrasound shifted diffraction peaks from 10.53° to 9.16° with increased intensity, reflecting changes in non-covalent interactions [120]. Thermal analysis demonstrated that ultrasound-treated pea protein isolate-high methoxyl pectin conjugates maintained structural integrity at temperatures up to 75 °C [78], confirming enhanced thermal stability. Microscopic observations by SEM, TEM, and AFM consistently confirmed that ultrasound yields smaller, more uniformly distributed nanoparticles with denser and more homogeneous microstructures. AFM revealed that zein-GA-resveratrol nanoparticles transformed from irregular, aggregated particles (height 193.4 nm, Ra 15.20 nm) into uniformly dispersed spherical nanoparticles (height 107.8 nm, Ra 8.67 nm) [119]. AFM also confirmed that CPP-chitosan-quercetin nanoparticles broke large aggregates, reducing particle height from 158.1 nm to 103.2 nm [120]. Similarly, TEM and SEM confirmed that zein- chitosan- resveratrol nanoparticles transformed from rough, irregular surfaces to smooth spherical morphologies [5].

These findings collectively demonstrate that ultrasound serves as a structural regulator orchestrating the multi-scale organization of protein-polysaccharide-bioactive systems, from molecular interactions (hydrogen bonding, electrostatic forces) to secondary structure order (α-helix enrichment) and microstructural uniformity (smooth spherical morphology). This hierarchical structural regulation directly enhances encapsulation efficiency, bioactivity retention, and sustained release performance. Notably, bioactive compounds can also be delivered through other forms of protein-polysaccharide complexes beyond nanoparticles, including emulsions (Section 5.1), gels (Section 5.2), and films (Section 5.3), each offering distinct advantages depending on the target application and the nature of the bioactive compound. However, the biological fate of these delivery systems,including targeted release and in vivo bioavailability, requires rigorous validation through animal studies, and the impact of ultrasound-induced free radicals on oxygen- or heat-sensitive bioactives warrants careful evaluation.

## 6 Conclusion

This review has systematically elucidated the role of ultrasound in modulating protein-polysaccharide interactions. Acoustic cavitation and its associated mechanical and chemical effects promote both covalent and non-covalent complexation by inducing protein unfolding, polysaccharide depolymerization, and exposure of reactive groups. These structural modifications enhance solubility, emulsification, and foaming properties, and have expanded the applications of the complexes in emulsions, gels, edible films, and bioactive delivery systems. Collectively, the evidence establishes ultrasound as a structural regulator that orchestrates multi-scale organization from molecular interactions to macroscopic material properties.

## 7 Perspectives

Despite considerable progress, translating laboratory findings into industrial practice requires a strategic reorientation. Four critical imperatives should guide future research:

(1) Establishing quantitative correlations between ultrasonic parameters and molecular outcomes. Most current studies optimize ultrasound conditions through empirical trial and error, yielding results that are highly system-specific. Future work should systematically investigate how power density, frequency, treatment time, and mode influence protein unfolding and polysaccharide chain scission. This requires establishing comprehensive datasets that link ultrasonic conditions to key structural descriptors, which can then be used to develop predictive models for different biopolymer systems. This effort should be supported by *in situ* analytical tools, such as fluorescence spectroscopy and rheometry, coupled with machine learning approaches to accelerate the identification of predictive rules.

(2) Addressing reactor engineering and scale-up challenges. Laboratory-scale ultrasound probes operate on small volumes with heterogeneous energy distribution. Scaling up to continuous-flow industrial systems requires innovative reactor designs, such as multi-transducer arrays and flow-through cells, that ensure uniform cavitation across large volumes. The use of computational tools to predict cavitation field distribution prior to fabrication could significantly accelerate the design of effective large-scale reactors. Energy efficiency must also be critically evaluated through life-cycle assessment, and strategies like pulsed ultrasound and multi-frequency operation should be explored to enhance cavitation yield per unit energy input.

(3) Evaluating the safety and quality of ultrasound-modified complexes. Ultrasound-induced protein unfolding may alter allergenic potential, and accelerated Maillard reactions could generate advanced glycation end-products with unknown health implications. Furthermore, functional advantages demonstrated in model systems must be validated in complex food matrices. Comprehensive safety assessment should include digestibility studies, monitoring of AGEs formation, and sensory evaluation to ensure consumer acceptance. Systematic toxicological screening, sensory evaluation, and long-term stability studies are essential before commercial adoption.

(4) Characterizing the structural heterogeneity and batch-to-batch consistency of conjugates. Most studies rely solely on grafting degree as a metric of reaction efficiency, leaving the precise molecular architecture of conjugates largely uncharacterized. Advanced analytical platforms capable of identifying glycation sites, quantifying conjugation stoichiometry, and determining molecular weight distribution should be employed to provide a more complete structural picture. Routine characterization of multiple independently prepared batches is necessary to establish consistency standards for quality control and regulatory approval.

Looking ahead, ultrasound should be viewed not merely as a processing aid but as a design tool for the rational engineering of sustainable food structures. Its convergence with emerging fields, including 3D food printing and sustainable packaging, presents exciting opportunities. Realizing this potential will require multidisciplinary collaboration and sustained efforts to bridge mechanistic understanding with industrial viability. By addressing the priorities outlined above, the field can fully leverage ultrasound to create nutritious, safe, and sustainable foods that meet evolving consumer and planetary demands.

## CRediT authorship contribution statement

**Qiufang Liang:** Writing – review & editing, Investigation, Funding acquisition, Conceptualization. **Panpan Cao:** Writing – original draft, Formal analysis, Data curation. **Yunxia Du:** Writing – review & editing, Formal analysis. **Haile Ma:** Supervision, Conceptualization. **Abdur Rehman:** Writing – review & editing. **Ka-Hing Wong:** Writing – review & editing. **Ren-You Gan:** Writing – review & editing. **Mingming Zhong:** Supervision, Investigation. **Yufan Sun:** Writing – review & editing. **Xiaofeng Ren:** Supervision, Funding acquisition.

## Declaration of competing interest

The authors declare that they have no known competing financial interests or personal relationships that could have appeared to influence the work reported in this paper.
